# Development of a short and universal learning self-efficacy scale for clinical skills

**DOI:** 10.1371/journal.pone.0209155

**Published:** 2019-01-07

**Authors:** Yi-No Kang, Chun-Hao Chang, Chih-Chin Kao, Chien-Yu Chen, Chien-Chih Wu

**Affiliations:** 1 Centre for Evidence-Based Medicine, Department of Education, Taipei Medical University Hospital, Taipei, Taiwan, Republic of China; 2 Department of Education and Humanities in Medicine, School of Medicine, College of Medicine, Taipei Medical University, Taipei, Taiwan, Republic of China; 3 Department of Math, Science and Technology, Teachers College, Columbia University, New York, United States of America; 4 Division of Nephrology, Department of Internal Medicine, Taipei Medical University Hospital, Taipei, Taiwan, Republic of China; 5 Department of Internal Medicine, School of Medicine, College of Medicine, Taipei Medical University, Taipei, Taiwan, Republic of China; 6 Department of Education, Taipei Medical University Hospital, Taipei, Taiwan, Republic of China; 7 Department of Anesthesiology, School of Medicine, College of Medicine, Taipei Medical University, Taipei, Taiwan, Republic of China; 8 Department of Anesthesiology, Taipei Medical University Hospital, Taipei, Taiwan, Republic of China; 9 Department of Urology, Taipei Medical University Hospital, Taipei, Taiwan, Republic of China; University of Florence, ITALY

## Abstract

**Background:**

Learning self-efficacy, defined as learners’ confidence in their capability to learn specific subjects, is crucial for the enhancement of academic progress, because it is positively correlated with academic achievements and effective learning strategy use. In this study, we developed a universal scale called the Learning Self-Efficacy Scale (L-SES) for Clinical Skills for undergraduate medical students and validated it through item analysis and content validity index (CVI) calculation.

**Design:**

The L-SES was developed based on the framework of Bloom’s taxonomy, and the questions were generated through expert consensus and CVI calculation. A pilot version of the L-SES was administered to 235 medical students attending a basic clinical skills course. The collected data were then examined through item analysis.

**Results:**

The first draft of the L-SES comprised 15 questions. After expert consensus and CVI calculation, 3 questions were eliminated; hence, the pilot version comprised 12 questions. The CVI values of the 12 questions were between .88 and 1, indicating high content validity. Moreover, the item analysis indicated that the quality of L-SES reached the qualified threshold. The results showed that the L-SES scores were unaffected by gender (*t* = −0.049; 95% confidence interval [−.115, .109], *p* > .05).

**Conclusion:**

The L-SES is a short, well-developed scale that can serve as a generic assessment tool for measuring medical students’ learning self-efficacy for clinical skills. Moreover, the L-SES is unaffected by gender differences. However, additional analyses in relevant educational settings are needed.

## Introduction

The statement “I can because I believe I can” reveals that mental confidence may influence the cognitive learning capabilities and perceived learning skills of an individual. This self-perception of confidence in the learning process or a learning strategy is often called “learning self-efficacy;” it reflects how confident a learner is about achieving specific learning goals in a particular learning context, process, or strategy [1, 2]. According to the concept of self-efficacy, learning self-efficacy can be defined as learners’ beliefs and confidence about their learning capabilities to produce given attainments [3]. Previous research has indicated that learners’ with high learning self-efficacy for their capabilities appear to set higher learning goals, persist for longer, and adapt more suitably to changes in the learning environment than do those with low learning self-efficacy [4]. In addition, high self-efficacy causes changes in the emotional states of learners because learners with high learning self-efficacy are less vulnerable to learning stress and are highly resilient to unfamiliar challenges [5, 6]. Therefore, understanding and investigating learners’ perceived learning self-efficacy are crucial because examining their mindset, mental pressure, motivation, persistence, and commitment when engaging in learning unfamiliar content can inform the design of curriculum and pedagogy.

The study of learners’ learning self-efficacy is considerably complex in medical education settings because numerous unpredictable variables influence real-world clinical decision-making [7]. The accuracy of clinical assessment greatly depends on not only the professional knowledge of doctors but also their mental confidence to effectively monitor the learning process and tease out the complexity of different clinical cases. Therefore, learning self-efficacy has become a core construct that medical students are required to develop, particularly in the practice of clinical medicine. However, relevant studies have shown that medical majors often struggle with transferring clinical skills gained from experiences in simulated laboratory settings to clinical settings [8]. Therefore, effectively understanding and evaluating medical students’ perceived self-efficacy for addressing unforeseen challenges in everyday clinical practices are crucial.

Studies of medical students’ learning self-efficacy have shown that their perceived self-efficacy and clinical performance are closely related [9, 10]. Researchers have developed and implemented different assessment scales to assess medical students’ self-efficacy in the classroom [11]. However, many of this kind of studies have focused on a particular medical domain [12, 13] or a particular medical skill [14]; hence, their results are not generalizable to medical students’ learning self-efficacy. Owing to the lack of a comprehensive and applicable assessment scale that measures the overall learning self-efficacy of medical students in clinical practice, the development of a generic, universal scale that examines medical students’ learning self-efficacy for clinical skills is imperative.

In response to the two aforementioned requirements, in this study, we developed a short, universal assessment scale to measure the learning self-efficacy of undergraduate medical students. To gain a comprehensive understanding of students’ learning self-efficacy in clinical medicine, we incorporated an educational framework to guide the design of our self-efficacy scale. Because Bloom’s taxonomy is a popular and overarching framework in education [15–17]. It has been broadly applied in not only education, but also in histology, pathology, pharmacy, nursing, and other health care professional courses in recent years [18–24].

About the Bloom’s taxonomy, it was developed by Bloom and his colleagues since 1956(Bloom, 1956). They proposed this taxonomy by categorizing learning objectives into three core domains and revised it to be more practical and completed in 2001 (Anderson et al., 2001). In overall, they tried to manifest hierarchy and category of learning activity. The core categories involve the cognitive domain (i.e., mental skills), affective domain (i.e., emotions and feelings), and psychomotor domain (i.e., physical skills). Cognitive domain is about mental skill including knowledge, cognition, and the development of intellectual skills [15]. Affective domain is related to the emotion, feeling, and their development. In this domain, Bloom’s taxonomy focuses on how people deal with the thing emotionally especially in learning activity [16, 25]. Then, psychomotor domain is related to physical skill and its development [17]. In educational practice, Bloom’s taxonomy should be considered as a framework of goals setting for teaching and learning. That is to say, a teaching or learning should have knowledge goal, skill-based goal and affective goal [15]. Although many healthcare professional fields applied this complete framework in their teaching and learning, many of them targeted on cognitive domain only [18, 19, 21, 22]. Besides, no learning self-efficacy scale in medical education was developed according to this framework, and the most efficacy scales in medical education were developed for a specific domain or a particular skill [12–14]. To fulfill the needs of teaching and learning evaluation among the diverse clinical skills, therefore, our study aimed to propose a universal scale to measure the learning self-efficacy of clinical skill according to the Bloom’s taxonomy.

## Methods and materials

In this study, we developed a scale called the Learning Self-Efficacy Scale (L-SES) for clinical skills for assessing medical students’ learning self-efficacy for clinical skills. The development of the L-SES comprised two steps, and it was examined by item analysis. In the first step, the first draft of the L-SES was developed based on theories of both self-efficacy and Bloom’s taxonomy of educational objectives. In the second step, the expert panel method was used, and the content validity index (CVI) was calculated. The pilot version of the L-SES was generated after an expert panel survey, and this version was administered to medical students attending a basic clinical skills course. To assess the quality of the L-SES, the investigator analyzed medical students’ data.

### Participants

Two groups of participants were enrolled in this study. The first group consisted of eight experts from different disciplines, comprising two physician clinical teachers, two nursing clinical teachers, two medical education professors, and two professors with expertise in education and assessment. They formed the expert panel in the L-SES development phase. The two physician clinical teachers were directors of the center for clinical skills. The two nursing clinical teachers participated in clinical skills instruction. The second group consisted of 235 fourth- or fifth-year medical students. They filled the questionnaire, and their data were used to examine the quality of the L-SES.

### Development steps

**Fig 1:**
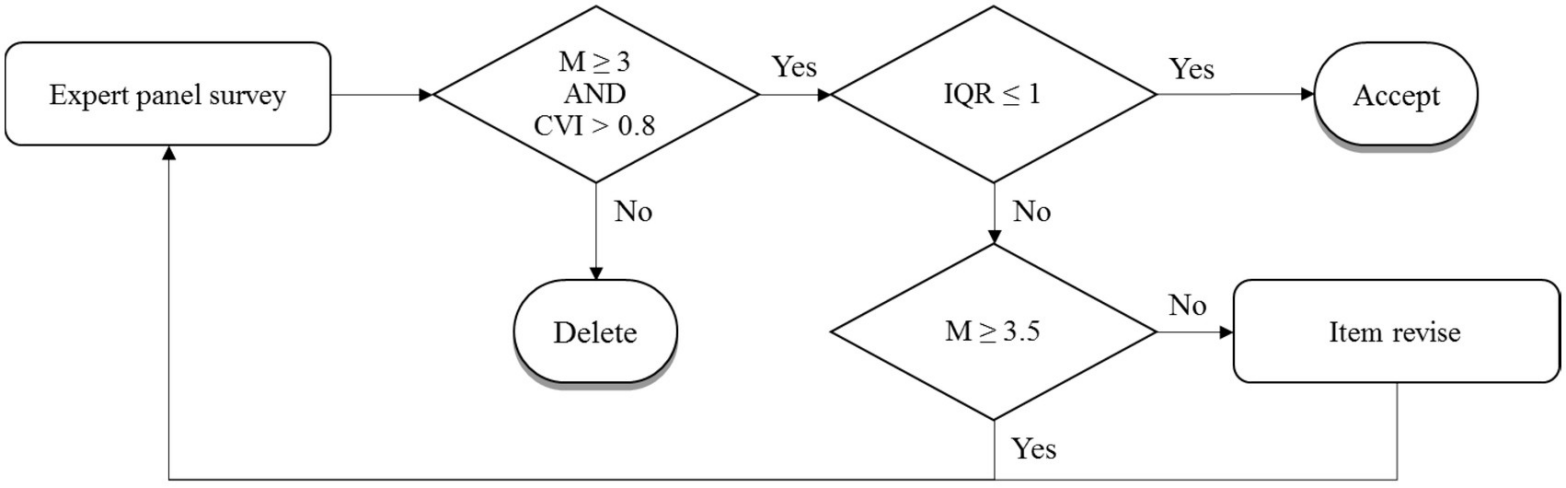
Criteria and flow for expert panel method. *M*, Mean; *IQR*, Interquartile range.

The L-SES was developed in two phases (questionnaire drafting and quality assessment phases), with a total of eight steps. The first phase consisted of five steps, and the second phase consisted of three steps. The five steps in the questionnaire drafting phase were as follows:

1. Drafting the L-SES expert evaluation version according to relevant self-efficacy literature and the Bloom’s taxonomy of educational objectives
2. Setting criteria for expert invitation
3. Setting criteria and flow for expert panel method (Fig 1)
4. Conducting an expert panel survey by using the L-SES expert evaluation version
5. Editing the L-SES pilot version according to the expert panel survey results

The professional teaching and research experience of the experts were used as the criteria for expert invitation. For example, experts were invited if they had taught clinical skills for >3 years, published medical education research papers (first author or corresponding author) within 1 recent year, or had recently conducted empirical studies related to scale development or self-efficacy. The invited experts evaluated the L-SES expert evaluation version using a 4-point Likert scale (1, 2, 3, and 4 indicate that the item should be deleted, requires major revision, requires minor revision, and is suitable for use, respectively). The investigators calculated CVI values from expert panel survey. The L-SES pilot version was developed according to the structured evaluation and opinions from experts, and it was designed on a 4-point Likert scale. The quality assessment phase comprised the following three steps:

1. Administering the L-SES pilot version to medical students for data collection
2. Setting the criteria for item analysis with reference to statistical rules
3. Conducting item analysis

The following criteria were used for item analysis: the *t* value of a critical ratio should be >3.5 with a statistically significant difference (*p* < .05, 95% confidence interval does not cross 0), item-total correlation and corrected item-total correlation should be >.40, Cronbach’s α of the entire scale should be >.70, and if an item is deleted, Cronbach’s α should be lower than Cronbach’s α of the entire scale.

### Data analysis

Microsoft Excel 2010 was used to calculate the mean (*M*), interquartile range (*IQR*), and CVI values from the data of the expert panel survey. Moreover, we examined the ratings provided by experts with different backgrounds by using the Kruskal—Wallis test. Using the Statistical Package for Social Sciences, Version 19, item analysis was conducted using the data of the survey of the L-SES pilot version. The item analysis involved the calculation of critical ratio, item-total correlation, and Cronbach’s α. The critical ratio was used for evaluating the discrimination between high-score and low-score groups of responses. The high-score group and the low-score group were selected from the top and bottom one-third, respectively, of the responses that were sorted by using the individual sum score of the L-SES in the descending or ascending order. Item-total correlation was used to examine the correlation between each item and the total score [26]. The coefficient of item-total correlation should be >.30 [27]. Cronbach’s α is used for testing reliability [28]. Cronbach’s α coefficient should be >.70. The statistical differences in this study was judged when p value was lower than 0.05 or 95% confidence interval (CI) did not cross value “0”.

### Ethical consideration

The study was a part of the project of the Ministry of Science and Technology Taiwan, and it was reviewed by Taipei Medical University—Joint Institutional Review Board (N201602094). The Taipei Medical University—Joint Institutional Review Board approved the study protocol on October 27, 2016.

## Results

### Questionnaire drafting of the L-SES

The L-SES expert evaluation version was developed based on the theories of both learning self-efficacy and Bloom’s taxonomy of educational objectives and consisted of 15 questions. This version also comprised three domains of clinical skills self-efficacy: the cognitive, affective, and psychomotor domains, which comprised six, four, and five questions, respectively. However, of the 15 questions, three were excluded according to the results of the expert panel survey. Two of the three questions were from the cognitive domain, and their CVI values were < .80. The third deleted question was from the affective domain, and its CVI value was < .75, which did not meet the threshold (Table 1). All the questions that passed the criteria (CVI > .80, *M* ≥ 3.00, and *IQR* ≤ 1) were amended and included in the L-SES pilot version according to the results of the panel survey and opinions of the experts.

**Table 1:** Expert content validity index values of 12 questions in the L-SES.

Item	Question	M	IQR	CVI	Judge
**Cognitive domain**					
C1-1	I can recall how to perform the clinical skill.	3.5	0.25	0.88	Accept
C1-2	I understand the content of the clinical skill and can demonstrate it to others.	3.5	1	0.88	Accept
C1-3	In case I forget the steps to operate the clinical skill, I can figure things out through reasoning.	3.25	1.25	0.67	Delete
C1-4	I can masterfully operate the clinical skill.	2.875	3	0.75	Delete
C1-5	I can verbally explain the purpose and principle of operating the clinical skill.	4	0	1	Accept
C1-6	I can verbally explain the sequence and interrelationship between each step.	4	0	1	Accept
**Affection domain**					
A2-1	I think I spend more time on this course than on others.	4	0	1	Accept
A2-2	I think I gain more in this course than in others.	3.875	0	1	Accept
A2-3	I tend to pay more attention to information related to this course.	4	0	1	Accept
A2-4	I tend to actively look for information related to this course.	3.5	0.25	0.88	Accept
**Psychomotor domain**					
P3-1	I can precisely imitate the instructor’s steps and actions of the clinical skill.	3.5	1	1	Accept
P3-2	I can smoothly complete the operation steps of the clinical skills.	3.625	1	1	Accept
P3-3	I try to monitor my clinical skill for improvements.	3.25	1	0.88	Accept
P3-4	I try to operate the clinical skill through different approaches.	3	1.5	0.75	Delete
P3-5	I try to monitor my clinical operations and make proper adjustments as needed.	3.5	1	0.88	Accept
Criteria		≥ 3	≤ 1	> 0.8	

*M*, Mean; *IQR*, Interquartile range

Additional analysis of the expert panel survey results confirmed that no differences were observed in the question quality evaluation among physician clinical teachers, nursing clinical teachers, medical education professors, and education professors. According to the Kruskal—Wallis test, no differences were observed in the items and domains among the experts with different backgrounds (Table 2). The mean ranks varied between 1.75 and 6.50, and the chi-square values varied between 0.000 and 6.857. All *p* values were >0.05.

**Table 2:** Background differences in the L-SES (Kruskal—Wallis test).

	Clinical teachers		Medical education	Education	Kruskal Wallis test	
Domain/Item	Physicians ^a^	Nurses ^a^	professors ^a^	professors ^a^	*χ*^2^	*p*
**Cognitive**	6.50	4.50	5.25	1.75	4.651	.199
C1-1	5.50	5.50	5.50	1.50	6.857	.077
C1-2	6.00	3.50	4.25	4.25	1.500	.682
C1-5	4.50	4.50	4.50	4.50	0.000	1.000
C1-6	4.50	4.50	4.50	4.50	0.000	1.000
**Affective**	5.50	3.25	5.50	3.75	2.357	.502
A2-1	4.50	4.50	4.50	4.50	0.000	1.000
A2-2	5.00	5.00	5.00	3.00	3.000	.392
A2-3	4.50	4.50	4.50	4.50	0.000	1.000
A2-4	5.50	3.25	5.50	3.75	2.357	.502
**Psychomotor**	6.50	5.25	3.75	2.50	3.500	.321
P3-1	6.50	4.50	4.50	2.50	3.500	.321
P3-2	6.00	4.00	6.00	2.00	5.133	.162
P3-3	6.50	4.75	3.75	3.00	2.750	.432
P3-5	6.00	6.00	3.50	2.50	4.222	.238

^a.^ mean rank

### Quality assessment of the L-SES

The quality of the L-SES was assessed based on the responses of 235 medical students attending a basic clinical skills course. The L-SES showed good item discrimination, unitary ability, and reliability (Table 3).

**Table 3:** Results of item analysis of 12 questions in the entire L-SES.

	Critical ratio		Item-total correlations		Cronbach’s α
Item	*t*-value	*95% CI*	Original	Corrected	if item deleted
C1-1	13.450*	[0.769 to 1.033]	.761	.707	.925
C1-2	20.579*	[0.833 to 1.009]	.776	.730	.924
C1-5	19.038*	[0.852 to 1.049]	.774	.726	.924
C1-6	21.193*	[0.855 to 1.030]	.808	.768	.923
A2-1	12.194*	[0.830 to 1.151]	.721	.647	.928
A2-2	18.283*	[0.848 to 1.053]	.728	.668	.926
A2-3	14.779*	[0.797 to 1.043]	.755	.699	.925
A2-4	12.216*	[0.745 to 1.032]	.730	.667	.927
P3-1	11.719*	[0.687 to 0.966]	.745	.690	.926
P3-2	16.190*	[0.857 to 1.094]	.822	.780	.922
P3-3	17.608*	[0.713 to 0.893]	.695	.640	.927
P3-5	24.175*	[0.840 to 0.989]	.773	.728	.924
Criteria	> 3.5	Do not cross 0	> .40	> .40	< .931

* *p* < .05. 95% CI: 95% confidence interval

In the item discrimination of the L-SES, the *t* values for the 12 questions of the L-SES varied between 11.719 and 24.175, with statistical significance (*p* < .001). The *t* values for the cognitive domain of the L-SES varied between 13.450 and 21.193, indicating high discrimination. The *t* values for the affective domain of the L-SES varied between 12.194 and 18.283, indicating high discrimination. The *t* values for the psychomotor domain of the L-SES (which comprised four questions) varied between 11.791 and 24.175, indicating high discrimination. All the 95% confidence intervals (95% CI) were > 0 and did not cross 0. All the *p* values for discrimination were < .001. These results indicated that the L-SES had high discrimination. The coefficients of item-total correlations varied between .695 and .822 for the entire L-SES, and the coefficients of corrected item-total correlations varied between .640 and .780 for the entire L-SES. All four questions in the cognitive domain of the L-SES varied between.761 and .808, and the coefficients of corrected item-total correlations varied between .707 and .768. For the questions in the affective domain of the L-SES, item-total correlations (*r*) ranged between .721 and .755. The corrected item-total correlations (*r*) of the affective domain ranged between .647 and .699. Four questions in the psychomotor domain exhibited item-total correlations (*r*) between .695 and .822. The corrected item-total correlations (*r*) of the psychomotor domain were from .640 to .780.

Regarding the reliability of the L-SES, Cronbach’s α coefficient was .931 for the 12 questions. Cronbach’s α coefficients varied between .922 and .928 when each question was deleted. The reliability of the L-SES was high, because all Cronbach’s α coefficients were lower than .931 when each question was deleted.

This study examined gender differences in L-SES scores. The results showed that the scores were unaffected by gender (Table 4). No difference was observed in the L-SES total score between the male and female participants (*t* = −0.049; 95% CI [−.115, .109], *p* > .05). The learning-self—efficacy-for-clinical-skills scores in the cognitive domain were similar between the male (*M* = 13.140; standard deviation *[SD]* = 1.822) and female (*M* = 13.242; *SD* = 1.885) participants (*t* = −0.421; 95% CI [−.584, .379], *p* > .05). The scores in the affective domain of the L-SES were similar between the male (*M* = 13.007; *SD* = 2.006) and female (*M* = 12.939; *SD* = 2.009) participants (*t* = 0.256; 95% CI [−.454, .590], *p* > .05). The scores in the psychomotor domain of the L-SES were similar between the male (*M* = 13.294; *SD* = 1.683) and female (*M* = 13.293; *SD* = 1.808) participants (*t* = 0.005; 95% CI [−.451, .453], *p* > .05).

**Table 4:** Gender differences in L-SES scores.

	Male		Female				
Variable	M	SD	M	SD	*t*-value	*95% CI*	*p*
Cognitive domain	13.140	1.822	13.242	1.885	-.421	[-.584, .379]	.674
Affective domain	13.007	2.006	12.939	2.009	.256	[-.454, .590]	.798
Psychomotor domain	13.294	1.683	13.293	1.808	.005	[-.451, .453]	.996
Total	3.287	0.420	3.290	0.442	-.049	[-.115, .109]	.961

*M*, Mean; *SD*, standard deviation; 95% CI, 95% confidence interval.

## Discussion

In this study, we developed the L-SES, which is a short but well-developed and verified scale that can serve as a generic assessment tool for understanding the relationship between medical students’ learning self-efficacy and practice of clinical skills. Bloom’s taxonomy of learning objectives offered a well-organized, overarching framework that guided the design and implementation of this scale. Because learning self-efficacy is positively correlated with academic achievements and effective learning strategy use, a comprehensive investigation of learning self-efficacy is crucial for the enhancement of academic progress [2]. The L-SES is the first universal tool for the measurement of learning self-efficacy for clinical skills. This tool differs from tools developed used in previous studies [29–31]. The objective of the previous tools was to measure the confidence of medical students in domain-specific clinical skills or clinical performance; therefore, they cannot be used to examine how confident medical students are when clinical practices are treated as a general skillset. Because the items of the previous tools were context-specific, the tools cannot be easily used in measurements of different clinical skills.

The three questions deleted based on the expert panel survey results (Table 1) were worth exploring. Item C1-4 was considered unsuitable because for students to “masterfully use clinical skills,” they require skills not only in the cognitive dimension but also in the affective and psychomotor dimensions. Hence, including this question in the cognitive domain was unsuitable. Item C1-3 was removed because of its tone of voice; a conditional sentence, rather than a declarative sentence, was used. The experts suggested that conditional sentences in scale items should be avoided to maintain consistency. The deleted item C3-4 implied that the practice of clinical skills should follow specific rules of conduct and should not be flexible for accommodating different approaches. All of the above opinions from the panel experts were crucial to the improvements and development of the L-SES.

Regarding gender differences in self-efficacy, the results of previous studies varied according to the objects of self-efficacy [32–35]. For instance, male participants may have higher self-efficacy in the use of information and communication technology and associated learning than female participants [34, 35]. In the present study, L-SES scores did not differ between the male and female participants. This result is similar to that of a previous study that developed of a self-efficacy scale [33], and it shows that quality of the L-SES is unaffected by gender.

## Conclusions

From the data analysis results, the L-SES was proven to be an internally consistent and reliable scale for measuring medical students’ learning self-efficacy for clinical skills. In addition, opinions from the panel experts and feedback from the undergraduate students confirmed the suitability of the L-SES. This learning self-efficacy scale was unaffected by gender differences. The L-SES was created in this study in response to the need for a generic, universal learning self-efficacy scale that can be applied to a broad spectrum of clinical medicine rather than domain-specific learning tasks [13, 36]. For example, the final version of the L-SES can be easily implemented in relevant studies by replacing the quoted phrases with target clinical skills (Table 5). Follow-up studies are necessary to investigate the clinical utility of the L-SES and how to monitor medical students’ learning of clinical skills through the L-SES.

**Table 5:** The final version of the L-SES.

Domain/No.	Disagree <----------------> agree					
	Item	1	2	3	4	5
**Cognitive**						
1	I can recall how to perform “the clinical skill”.	1	2	3	4	5
2	I understand the content of “the clinical skill” and can demonstrate it to others.	1	2	3	4	5
3	I can verbally explain the purpose and principle of operating “the clinical skill”.	1	2	3	4	5
4	I can verbally explain the sequence and interrelationship between each step.	1	2	3	4	5
**Affective**						
5	I think I spend more time on “this” course than on others.	1	2	3	4	5
6	I think I gain more in “this” course than in others.	1	2	3	4	5
7	I tend to pay more attention to information related to “this” course.	1	2	3	4	5
8	I tend to actively look for information related to “this” course.	1	2	3	4	5
**Psychomotor**						
9	I can precisely imitate the instructor’s steps and actions of “the clinical skill”.	1	2	3	4	5
10	I can smoothly complete the operation steps of “the clinical skill”.	1	2	3	4	5
11	I try to monitor my “clinical skill” for improvements.	1	2	3	4	5
12	I try to monitor my “clinical” operations and make proper adjustments as needed.	1	2	3	4	5

Users can replace the quoted phrases with target clinical skills

## Abbreviations

CI: confidence interval
CVI: content validity index
IQR: interquartile range
L-SES: Learning Self-Efficacy Scale
M: mean
SD: standard deviation
